# Response-time process signals for selective escalation in reduced-item mental health symptom screening

**DOI:** 10.3389/fpsyt.2026.1926874

**Published:** 2026-08-25

**Authors:** Zidong Zhou, Cheng Liu, Xuanhe Wang

**Affiliations:** 1 School of Digital Arts, Jiangsu Vocational Institute of Commerce, Nanjing, China; 2 School of Digital Business, Jiangsu Vocational Institute of Commerce, Nanjing, China; 3 School of Computer Science and Engineering, Sun Yat-sen University, Guangzhou, China

**Keywords:** high-confidence disagreement, mental health screening, questionnaire score categories, reduced-item assessment, response time, response-process evidence, selective escalation, selective prediction

## Abstract

**Introduction:**

Reduced-item mental health symptom questionnaires can reduce respondent burden in large-scale screening, but some provisional results may still require confirmation through completion of the remaining items. We examined whether item-level response time (RT) could improve selective escalation after a 19-item screen.

**Methods:**

Using fixed-order data from 24,292 students who completed the PHQ-9, GAD-7, PSS-14, and ISI with item-level RTs, we conducted an offline participant-specific adaptive replay. Complete-questionnaire score categories served as the reference for evaluating the provisional categories issued after the 19-item screen.

**Results:**

Although RT did not materially improve overall score-category classification, it improved the prioritization of provisional decisions at risk of high-confidence disagreement (HCD) with complete-questionnaire categories. RT-based calibration also modestly reduced calibration error relative to the response-only kernel baseline, while standard isotonic calibration performed best. Among 14,576 scale-level test decisions, 579 met the operational definition of HCD. The confidence-plus-RT model achieved an average precision of 0.772, compared with 0.540 for the confidence-only model and 0.491 for the shuffled-RT control. At a 5% participant-escalation capacity, the confidence-plus-RT model captured 30.6% of HCD decisions, compared with 15.2% using confidence alone; at 10% capacity, the corresponding capture rates were 56.1% and 32.8%. These capacities corresponded to average questionnaire burdens of 19.9 and 20.8 items per participant, respectively, compared with 37 items under universal completion. The HCD-ranking advantage was observed across alternative confidence thresholds and item-selection designs, with the largest gain for PSS-14.

**Discussion:**

The results show that RT can serve as a complementary response-process signal for directing limited full-questionnaire completion capacity toward provisional decisions with the highest estimated HCD risk. Prospective, questionnaire-specific studies should evaluate this workflow in independent samples and live screening settings.

## 1 Introduction

Large student mental health screening programs require brief assessments and clear pathways to further care (1). Short questionnaires reduce respondent burden and may approximate longer measures at the group level, but some participant-level score-category decisions remain uncertain (2). The operational question is which reduced-item results can be accepted and which should trigger completion of the remaining items.

Administering the Patient Health Questionnaire-9 (PHQ-9), Generalized Anxiety Disorder-7 (GAD-7), 14-item Perceived Stress Scale (PSS-14), and Insomnia Severity Index (ISI) requires 37 items. Fixed short forms such as PHQ-2, GAD-2, and PSS-4 provide simple burden-reduction strategies (3–5). Conventional computerized adaptive tests based on item response theory combine sequential item selection with fixed-length or precision-based stopping rules (6). Data-driven adaptive assessment systems and machine-learning decision rules have also been developed (7–9). We evaluated a downstream two-stage workflow consisting of a participant-specific 19-item screen followed by capacity-constrained completion of the remaining items. The 19-item budget was imposed as a burden constraint rather than as a precision-based stopping criterion; the resulting score-category decisions were therefore treated as provisional. A decision-audit model then prioritized participants with the highest estimated risk of high-confidence disagreement (HCD). Together, these two stages link reduced respondent burden to the explicit allocation of limited follow-up capacity in large-scale screening. Because responses and response times (RTs) were collected under the original fixed administration order, the workflow was evaluated through offline participant-specific adaptive replay rather than prospective adaptive administration. A strict observed-first-19 design was examined separately as a sensitivity analysis.

Within this workflow, RT provides a candidate response-process signal for decision auditing. Lognormal, hierarchical, joint, and mixture models show that item duration can reflect item demands and heterogeneous response states (10–14). Careless responding can distort the reliability and validity of questionnaire scores (15–17). Previous analyses of the source dataset examined careless responding across mental health questionnaires and RT sequences during insomnia assessment (18, 19). Digital questionnaire RT is readily available as paradata, although it can also reflect cognitive, demographic, contextual, and questionnaire-specific influences (20). Accordingly, RT was treated as an operational response-process signal rather than as a direct measure of effort, ability, diagnosis, or symptom severity.

Selective prediction provides the decision framework. A system can defer a prediction when additional evidence is warranted, reducing disagreement among accepted decisions at the cost of lower coverage (21–23). Biomedical selective-prediction work similarly treats abstention as a way to improve reliability while controlling the burden of human review (24). In reduced-item screening, deferral means completing the remaining questionnaire items so that the complete-questionnaire score category replaces the provisional category. We tested whether selected-item RT summaries improved escalation ranking beyond model confidence.

We analyzed public item-level response and RT data from 24,292 students (25, 26). The PHQ-9, GAD-7, PSS-14, and ISI are rule-scored symptom questionnaires (27–30). The PHQ-9 and GAD-7 are screening instruments whose diagnostic accuracy varies across populations (31–33). We therefore treated all complete-questionnaire categories as score-derived reference groups rather than clinical diagnoses. We first evaluated classification and calibration, then examined the distribution and ranking of operational HCD events, the effect of escalation capacity, and robustness across preprocessing choices, comparator feature sets, item-selection designs, thresholds, and held-out scales. Shuffled-RT controls, calibration benchmarks, and leakage checks were used to assess the specificity and robustness of the audit signal.

The study addressed four research questions. RQ1: How often did the 19-item screen disagree with complete-questionnaire categories, and how were HCD and elevated-score false-negative (ES-FN) events distributed across scales? RQ2: Did RT improve HCD ranking relative to the same confidence model without RT and to shuffled-RT controls? RQ3: How did the incremental HCD capture associated with RT change across escalation capacities? RQ4: Were the findings robust to alternative preprocessing, thresholds, comparator feature sets, item-selection designs, and held-out-scale tests?

## 2 Materials and methods

### 2.1 Dataset, labels, and ethics

Participants were the unit of analysis, and the released data files were linked using the export_id field. Participants were divided into mutually exclusive training, validation, and test sets comprising 70%, 15%, and 15% of the sample, respectively. Stratification was based on the number of scales meeting the development-defined elevated-score threshold according to complete-questionnaire scores. The same participant split was retained throughout preprocessing, model fitting, model selection, and final test evaluation.

This secondary analysis used publicly available, anonymized data and involved no new recruitment, intervention, or participant contact. The Ethics Committee of Xinxiang Medical College of Henan Province approved the original data collection (approval no. XYLL-2020235). All participants provided electronic informed consent before completing the survey, including consent to the use and sharing of their data for scientific research, as reported in the original dataset publication (26).

Reference score categories were derived from questionnaire score bands and the distributions supplied with the dataset. PHQ-9 categories were 0–4, 5–9, 10–14, 15–19, and ≥20. ISI categories were 0–7, 8–14, 15–21, and ≥22, following established scoring conventions and prior psychometric work (27, 29, 34). GAD-7 categories (0–4, 5–9, 10–13, 14–18, and ≥19) and PSS-14 categories (0–14, 15–28, 29–42, and ≥43) were retained solely to reproduce the groupings in the released dataset. The GAD-7 boundaries differ from the commonly used 5, 10, and 15 thresholds (30). The Perceived Stress Scale assesses perceived stress rather than a diagnostic disorder (28), and the factor structures of its 10-item and 14-item versions have been examined in a systematic review and meta-analytic confirmatory factor analysis (35). These dataset-defined categories were treated as score-group indices. Elevated-score indicators used moderate-or-higher PHQ-9 categories, the third-or-higher dataset-defined categories for GAD-7 and PSS-14, and the moderate-or-higher ISI symptom-score category. Released complete-questionnaire score columns defined the reference scores and categories. One released PSS-14 score differed from the value reconstructed as the sum of the 14 item codes minus 14. We retained the released score to avoid researcher-defined relabeling. **Table 1** summarizes the data checks and category distributions. **Supplementary Table 1** summarizes the full analysis workflow and its information boundaries.

**Table 1:** Sample characteristics, dataset-defined score categories, and data-validation checks.

Item	Value	Note
Participants	24,292	Complete cases in the released data files
Item-level records	898,804	Participants × 37 questionnaire items
Train/validation/test	17,004/3,644/3,644	Disjoint participant split
PHQ-9 distribution	16,886/6,090/957/262/97	Five conventional score categories
GAD-7 distribution	20,170/3,576/332/157/57	Five dataset-defined score categories
PSS-14 distribution	6,607/14,884/2,687/114	Four dataset-defined score categories
ISI distribution	22,090/1,922/242/38	Four conventional score categories
Score reconstruction	PSS-14: 1 mismatch	Released score retained as reference

### 2.2 Response-time preprocessing

Item-level RTs were preprocessed to reduce the influence of zero values and extreme durations. For each item i, the 0.5th and 99.5th percentiles of raw RT were estimated from the training set. RTs in all three splits were winsorized to these item-specific limits. Consistent with the use of logarithmic scales in lognormal RT modeling (10–12), the winsorized RTs were then transformed as shown in Equation 1.

(1) $$L_{pi}=\text{log}(1+RT_{pi}^{win})$$

where $RT_{pi}^{win}$ is the winsorized RT for participant *p* on item *i*.

For this analysis, two ordinary least squares regressions were used to residualize RT before constructing the operational process features. The primary content-adjusted model included item word count, within-scale position, global administration position, and reference-coded scale indicators, as shown in Equation 2:

(2) $$L_{pi}=\beta_{0}+\beta_{1}WC_{i}+\beta_{2}WPOS_{i}+\beta_{3}GPOS_{i}+\gamma^{T}SCALE_{i}+\epsilon_{pi}^{\left(C\right)}$$

Here, ${\text{WC}}_{i}$ is item word count, ${\text{WPOS}}_{i}$ is within-scale position, ${\text{GPOS}}_{i}$ is global administration position, and ${\text{SCALE}}_{i}$ is a reference-coded vector of scale indicators. These covariates represented measured reading burden, local order, cumulative administration position, and between-scale RT differences (10, 12). The response_conditioned sensitivity model, shown in Equation 3, additionally included the observed item response, age, and reference_coded demographic indicators:

(3) $$L_{pi}=\alpha_{0}+\alpha_{1}WC_{i}+\alpha_{2}WPOS_{i}+\alpha_{3}GPOS_{i}+\alpha_{4}X_{pi}+\alpha_{5}Age_{p}+\delta^{T}SCALE_{i}+\theta^{T}DEMO_{p}+\epsilon_{pi}^{\left(R\right)}$$

Here, γ and δ denote distinct scale-indicator coefficient vectors estimated separately in the content-adjusted and response-conditioned models, respectively. $X_{\text{pi}}$ is the observed item response, and ${\text{DEMO}}_{p}$ contains gender, education, smoking, and drinking indicators. Categorical levels, reference categories, and both regression models were estimated from training item records only. The fitted encoders, coefficients, and all training-derived preprocessing quantities were then applied unchanged to validation and test records. The regressions assumed additive linear associations with mean RT on the log(1+RT) scale. The regressions were used to construct process features without coefficient-level or causal interpretation.

For residualization specification m∈{C,R}, the raw residual was defined as shown in Equation 4.

(4) $$e_{pi}^{\left(m\right)}=L_{pi}-{\hat{L}}_{pi}^{\left(m\right)}$$

and the standardized residual was defined in Equation 5.

(5) $$z_{pi}^{\left(m\right)}=\frac{e_{pi}^{\left(m\right)}-{\bar{e}}_{train}^{\left(m\right)}}{s_{train}^{\left(m\right)}}$$

The content-adjusted standardized residual $z_{\text{pi}}^{\left(C\right)}$ was the primary RT process signal, and the response-conditioned residual $z_{\text{pi}}^{\left(R\right)}$ was retained for sensitivity analysis. In the sequential simulation, a residual became available only after its item had been selected and answered. Population standard deviations (SDs; divisor *N*) were used in the selected-item summaries; they were set to zero when only one item was available. The residuals were used as operational process features rather than as reliability, diagnostic, or latent-speed measures. The training-set $R^{2}$ values were 0.038 for the content-adjusted model and 0.057 for the response-conditioned model; the corresponding residual SDs were 0.647 and 0.641. Residual diagnostics are reported in **Supplementary Figure 1** and **Supplementary Table 2**.

### 2.3 Offline sequential reduced-item simulation

At each item budget, the offline algorithm selected items sequentially from the remaining pool after a four-item initialization. For the adaptive policies, the four-item initialization set contained the highest-priority item from each scale, with item priorities derived from the training data. For participant p at step t, let $O_{\text{pt}}$ denote the selected and answered items, $O_{\text{pst}}\subseteq O_{\text{pt}}$ denote the selected items from scale s, $N_{\text{pst}}\text{=|}O_{\text{pst}}|$, and $m_{s}$ denote the number of items in that scale. After initialization, all state variables were recalculated after each selection using $O_{\text{pt}}$ only. Responses and RTs from unselected items, and features derived from the complete response set, were unavailable to selection and prediction.

Person-mean proration is a commonly used but assumption-dependent approach for scoring partially observed multi-item scales (36). We used it as an operational approximation; the selected-item score estimate for scale s was defined in Equation 6.

(6) $${\hat{S}}_{pst}=m_{s}(\frac{1}{N_{pst}}\sum_{i\in O_{pst}}X_{pi})-14\;I(s=\text{PSS})$$

The subtraction of 14 reproduced the PSS-14 score transformation used in the released dataset and analysis files. The resulting estimate retained the original questionnaire total-score scale. During the sequential simulation, it was mapped to the same dataset-defined categories used for the corresponding complete-questionnaire score. Let $B_{s}$ denote the internal score-category boundaries for scale s. The distance to the nearest boundary was defined in Equation 7.

(7) $$d_{pst}=\underset{b\in B_{s}}{\text{min}}|{\hat{S}}_{pst}-b|$$

Scale coverage and the remaining coverage gap were defined in Equation 8.

(8) $$Q_{pst}=\frac{N_{pst}}{m_{s}},\;C_{pst}=1-Q_{pst}$$

The under-covered-scale indicator equaled one when scale s had no more selected items than the currently least represented scale, as specified in Equation 9.

(9) $$A_{pst}=I(N_{pst}\leq \underset{q\in S}{\text{min}}N_{pqt})$$

where S is the set of four scales. The response_only uncertainty term was as follows:

(10) $$U_{pst}^{\left(0\right)}=\frac{1}{1+d_{pst}}+C_{pst}+0.10SD(\{X_{pi}:i\in O_{pst}\})$$

In Equation 10, the SD term was the population standard deviation of the selected responses and was set to zero when only one item had been observed for a scale.

We derived an item-priority score from the training data by combining each item’s absolute correlation with the corresponding complete-questionnaire score-category index and a small contribution from response variance. For candidate item j belonging to scale s(j), the item-priority score was defined in Equation 11.

(11) $$I_{j}=|{\text{Cor}}_{\text{train}}(X_{\text{pj}},Y_{p,s(j)})|+0.05{\text{Var}}_{\text{train}}(X_{\text{pj}})$$

Here, $X_{\text{pj}}$ is participant p’s response to item j and $Y_{p,s(j)}$ is the complete-questionnaire score-category index for the corresponding scale. Correlation and variance were calculated across training participants using population moments. The resulting score provided an empirical ranking of candidate items within each scale, and the highest-ranked item served as that scale’s initialization item.

For the RT-aware policy, process consistency was calculated from the standardized content-adjusted residuals of selected and answered items. The fast-response fraction and long-pause fraction were defined in Equation 12.

(12) $$F_{pt}=\frac{1}{\left|O_{pt}\right|}\sum_{i\in O_{pt}}I(z_{pi}^{\left(C\right)}<-1),\;L_{pt}=\frac{1}{\left|O_{pt}\right|}\sum_{i\in O_{pt}}I(z_{pi}^{\left(C\right)}>1)$$

Residual variability was defined in Equation 13.

(13) $$V_{pt}=SD(\{z_{pi}^{\left(C\right)}:i\in O_{pt}\})$$

The operational response-process consistency score was then defined in Equation 14.

(14) $$R_{pt}=\text{clip }[1-0.35F_{pt}-0.20L_{pt}-0.08\text{min}(V_{pt},4),\;0.10,\;1.00]$$

This score summarized the consistency of the RT residual patterns across selected items. The RT-aware scale uncertainty was defined in Equation 15.

(15) $$U_{pst}=U_{pst}^{\left(0\right)}[1+0.25(1-R_{pt})]$$

For the response-only policy, $R_{pt}$ was set to 1 so that $U_{pst}=U_{pst}^{\left(0\right)}$.

For an unselected candidate item j belonging to scale s = s(j), candidate utility was defined as:

(16) $$U_{pjt}=I_{j}(1+U_{pst})+\lambda_{pt}C_{pst}+0.08A_{pst}-\rho_{pt}N_{pst}+\eta_{pt}I_{j}(1+A_{pst})$$

In Equation 16, $U_{\text{pjt}}$ denotes candidate utility and is distinct from the scale uncertainty $U_{\text{pst}}$. The final term is the high-priority confirmation contribution, and the term − $\rho_{\text{pt}}$ $N_{\text{pst}}$ is the within-scale redundancy penalty. For the RT-aware policy, the time-varying coefficients were defined in Equations 17–19.

(17) $$\lambda_{pt}=0.20+0.40(1-R_{pt})$$

(18) $$\rho_{pt}=0.015+0.020(1-R_{pt})$$

(19) $$\eta_{pt}=0.25(1-R_{pt})$$

Setting $R_{\text{pt}}$ = 1 for the response-only policy yielded $\lambda_{\text{pt}}$ = 0.20, $\rho_{\text{pt}}$ = 0.015, and $\eta_{\text{pt}}$ = 0. At each step, the remaining item with the largest utility was selected. Numerical ties were resolved using a deterministic participant–item-specific perturbation that did not depend on responses or RT. The utility function and its coefficients were fixed during model development before test-set evaluation, rather than estimated from a likelihood or Bayesian model.

The primary analysis used a 19-item budget. The 11- and 26-item budgets represented lower- and higher-burden conditions. Two fixed-design sensitivity analyses were also conducted. The first used the initial 19 items in the released administration order. The second used a *post hoc* near-balanced 19-item set with allocations of 5, 5, 5, and 4 items to PHQ-9, GAD-7, PSS-14, and ISI, respectively. Items were ranked within each scale according to the training-derived item-priority score ${\text{I}}_{\text{j}}$ and were then ordered by their released global positions. If a fixed design contained no item from a scale, the prediction function used that scale’s training-set mean score. Each design generated its own reduced-form decisions and downstream event set, so model comparisons were made within rather than across designs. **Supplementary Methods S1**; **Supplementary Table 3**, and **Supplementary Table 4** document the fixed coefficients and their sensitivity analyses.

A short-form-seeded fixed comparator began with 11 anchor items: PHQ-9 items 1–2, GAD-7 items 1–2, PSS-14 items 2, 4, 5, and 10, and ISI items 1–3. At the 19- and 26-item budgets, the remaining positions were filled using the training-derived item-priority ranking. We therefore describe this comparator as a seeded fixed subset rather than a validated composite short form.

### 2.4 Calibration controls, learned baselines, and metrics

To distinguish how RT entered the analysis pipeline, we evaluated three variants: no RT use, label-preserving confidence adjustment, and label-changing score adjustment. The response-only variant used response-only item selection and prorated selected-item scores. The RT calibration-only variant retained the response-only selected items and issued labels but changed probability dispersion using final-budget process consistency. The RT score-adjusted variant used RT-aware item selection, added 0.20 times the scale-specific mean content-adjusted residual to the prorated score, and applied process-consistency-dependent shrinkage toward the training-set scale mean. The shrinkage coefficient was the smaller of 0.35 and 0.40(1 − $R_{\text{pB}}^{*}$). $R_{\text{pB}}^{*}$ extended the process-consistency score in Equation 14 with a 0.05 posterior-entropy penalty from a training-only three-component diagonal-covariance Gaussian-mixture model fitted to selected-item summaries. Adjusted scores were clipped to the valid scale range before category assignment.

For each scale, a selected-item score estimate was converted to a probability distribution over score-derived categories using a normalized squared-distance kernel. If category k for scale s had a lower boundary $a_{\text{sk}}$ and upper-exclusive boundary $b_{\text{sk}}$, its center was defined in Equation 20.

(20) $$c_{sk}=\frac{a_{sk}+b_{sk}-1}{2}$$

For each participant and category, the unnormalized squared-distance weight and the corresponding normalized category probability were defined in Equations 21 and 22.

(21) $$w_{psk}=\exp\{-\frac{1}{2}{\left[\frac{{\hat{S}}_{ps}-c_{sk}}{\sigma_{s}}\right]}^{2}\}$$

(22) $$\text{P}(Y_{ps}=k|{\hat{S}}_{ps})=\frac{w_{psk}}{\sum_{h=1}^{K_{s}}w_{psh}}$$

For each policy, item budget, variant, and scale, $\sigma_{s}$ was selected on the validation split by minimizing expected calibration error and was then applied unchanged to the test split. For the label-preserving RT calibration-only control, participant-specific smoothing was defined in Equation 23.

(23) $$\sigma_{ps}^{\text{RT}}=\sigma_{s}[1+\gamma_{\text{RT}}(1-R_{pB})],\gamma_{\text{RT}}=1.25$$

where $R_{pB}$ is the Equation 14 process-consistency score at the final item budget. This adjustment changed probability dispersion without changing the response-only issued score-category label.

For label-preserving controls, label-aware expected calibration error (ECE-L) evaluated the calibrated probability assigned to the fixed response-only label. Argmax-based expected calibration error (ECE-A) allowed the calibrated distribution to determine the argmax label. Argmax-change rates were reported as diagnostics. Brier score, negative log-likelihood (NLL), ECE-L, and ECE-A were used to describe probabilistic performance (37–39). Macro-averaged F1 score (macro-F1), elevated-score recall, mean absolute error (MAE) in score-category index, and quadratic weighted kappa were used to describe classification and agreement (38, 40).

Calibration benchmarks included temperature scaling, one-vs-rest isotonic calibration, constant confidence softening, shuffled-RT placebo calibration, RT calibration-only adjustment, and RT score adjustment. Scale-specific smoothing values were selected on the validation split from the configured grid; boundary analyses extended the σ and temperature range to 10. Learned response-only comparators used 37 masked item responses and 37 binary selection indicators. The two models fixed during development were a class-balanced classification and regression tree (CART; maximum depth 6; minimum leaf size 40) and class-balanced L2-regularized one-vs-rest logistic regression with liblinear optimization. Both were fitted on the training split. Validation negative log-likelihood was recorded, and test performance was reported separately; neither model was selected using test results. Models were implemented in scikit-learn (41).

### 2.5 Short-form disagreement and high-confidence disagreement outcomes

For each participant and scale, the 19-item response-only screen generated one analysis record for decision auditing. Each row contained the complete-questionnaire category, short-form prediction, confidence assigned to the issued label, entropy, probability margin, distance to the nearest category boundary, scale coverage, selected-item count, and selected-item RT summaries. We defined three audit outcomes: any disagreement between the short-form and complete-questionnaire categories; ES-FN, in which the complete score met the scale-specific elevated threshold but the short form did not; and operational HCD.

The primary HCD threshold was the 75th percentile of issued confidence among validation-set disagreements. Let τ denote this validation-derived threshold. A decision was labeled HCD when the short-form category disagreed with the complete-questionnaire category and the issued confidence was at least τ. The same pooled threshold was applied unchanged to training and test audit rows. Alternative validation quantiles were examined as threshold sensitivities.

The same validation split was used to define the operational HCD threshold and to select audit-model hyperparameters. This dual use may have introduced development-stage optimism; the test split remained untouched until final evaluation.

Two additional analyses assessed sensitivity to the HCD definition. First, decisions were restricted to the fixed high-confidence gate, confidence≥τ, and disagreements versus agreements were modeled within that stratum. Second, the 75th-percentile threshold was estimated separately within validation disagreements for each scale. Scale-specific thresholds were evaluated with an inclusive rule ( ≥) and a strict-greater-than rule to quantify the effect of confidence ties at the threshold.

### 2.6 Selective escalation to questionnaire completion simulation

Selective escalation evaluated whether a decision-audit ranking could allocate additional questionnaire burden efficiently. All participants first completed the 19-item reduced screen. Participants in the highest-ranked 5%, 10%, 20%, or 30% were then selected to complete the remaining 18 items. For these participants, the four short-form scale decisions were replaced with the complete-questionnaire score-derived categories. After escalation, agreement was recalculated using the complete-questionnaire score categories that replaced the provisional categories. The average item burden was B(r)=19 + 18r, where r is the participant escalation fraction.

Scale-level audit scores were aggregated by taking the maximum score across the four scale decisions for each participant. One high-risk scale could therefore trigger completion of all remaining items. Random escalation was averaged over 200 draws. Other rankings included a lowest-confidence operational heuristic, an RT-only process score, supervised ordinary-disagreement and elevated-score-false-negative models, a shuffled-RT placebo, and an oracle reference based on test outcomes.

The target-matched HCD comparison used two otherwise identical supervised rankings: a confidence-only model and a confidence-plus-RT model. The models shared the outcome, model family, validation procedure, participant-level maximum aggregation, tie rule, and escalation capacities. Their only difference was the inclusion of RT summaries. The lowest-confidence heuristic was retained as an operational benchmark; it was not used to estimate the incremental contribution of RT.

### 2.7 Disagreement-detection models and negative controls

Audit models were fitted to training decision rows, tuned on the complete validation split, and evaluated once on the disjoint test split. The confidence-only feature set included issued confidence, entropy, and probability margin. Score-and-coverage features included boundary distance, selected-item count and fraction within each scale, and participant-level scale coverage. RT features included process consistency, fast-response fraction, long-pause fraction, residual SD, mean residual, and absolute mean residual. Scale was represented by one-hot indicators. The shuffled-RT placebo independently permuted the RT features while preserving all non-RT features.

All supervised audit models used a class-balanced L2-regularized logistic regression with liblinear optimization. C∈{0.1, 1.0, 10.0} was selected by validation average precision (AP). For computational tractability, the audit-training set was capped at 5,000 participants sampled with a fixed seed. The selected model was then refitted with this sample and all validation participants before test evaluation. Primary discrimination metrics were AP and the area under the receiver operating characteristic curve (ROC-AUC); top-capacity summaries reported precision and recall among the highest-ranked 10% of decision rows. Participant-level escalation used the capacities defined in Section 2.6 and deterministic tie-breaking by ascending participant identifier.

Scale-specific matched models were fitted separately within each scale, and an analysis excluding PSS-14 combined PHQ-9, GAD-7, and ISI. A response-rich sensitivity analysis added the exact 37-item selection mask and the corresponding masked responses to the confidence and score-and-coverage features. The matched response-rich-plus-RT model added the same six RT summaries. These analyses tested whether RT retained incremental information after detailed selected-response information was included.

Paired model differences were estimated by resampling participants with replacement and retaining all scale decisions from each sampled participant (42). Target-matched escalation, high-confidence-gated, and response-rich analyses used 500 resamples; scale-specific analyses used 300. Bootstrap resampling varied the test participants while keeping the fitted models, selected thresholds, and item-selection rules fixed. Further details on the target-matched HCD and design-sensitivity analyses are provided in **Supplementary Methods S2**.

This secondary observational analysis followed the Strengthening the Reporting of Observational Studies in Epidemiology statement. The Transparent Reporting of a multivariable prediction model for Individual Prognosis Or Diagnosis plus Artificial Intelligence statement informed reporting of the data source, participants, predictors, questionnaire score outcomes, data splitting, calibration, and evaluation. We used both frameworks as reporting guidance because the analysis was an offline questionnaire-score replay without a clinical-diagnosis reference standard (43, 44).

Process-quality strata were defined using thresholds estimated in the training set. Fast-inconsistent required both the fast-response fraction and residual SD to be at or above their 75th percentiles. Slow/interrupted required the long-pause fraction to be at or above the 75th percentile or the absolute mean residual to be at or above the 90th percentile. Stable-typical required process consistency to be at or above the 75th percentile, residual SD to be at or below the 25th percentile, and fast- and long-pause fractions to be at or below their medians. All other participants were assigned to the mixed-pattern stratum. These strata were calculated from RT summaries for the 19 items selected by the response-only policy.

## 3 Results

### 3.1 Classification and calibration benchmarks

**Figure 1:**
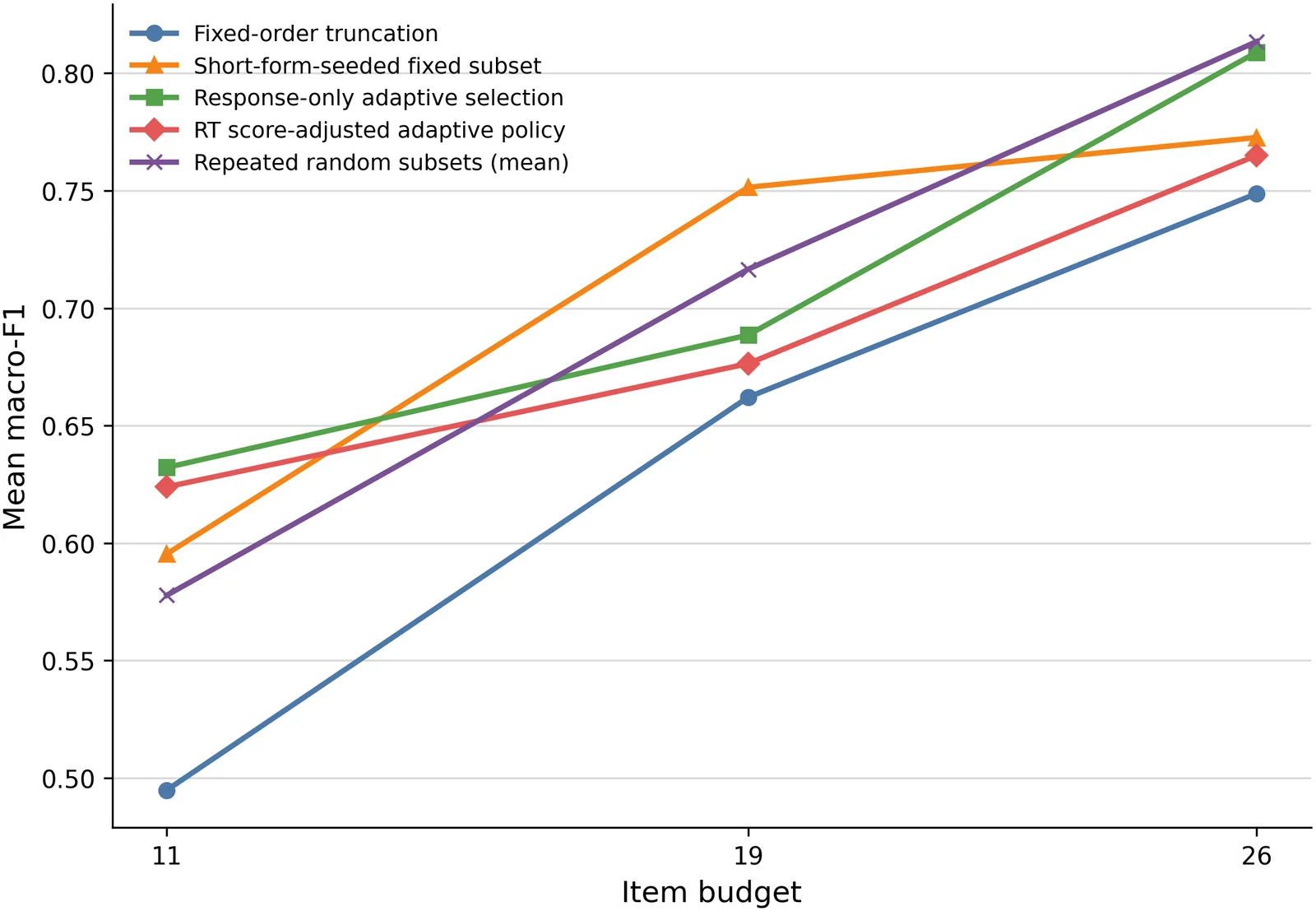
Mean macro-F1 by item budget and reduced-item analysis variant.

**Figure 2:**
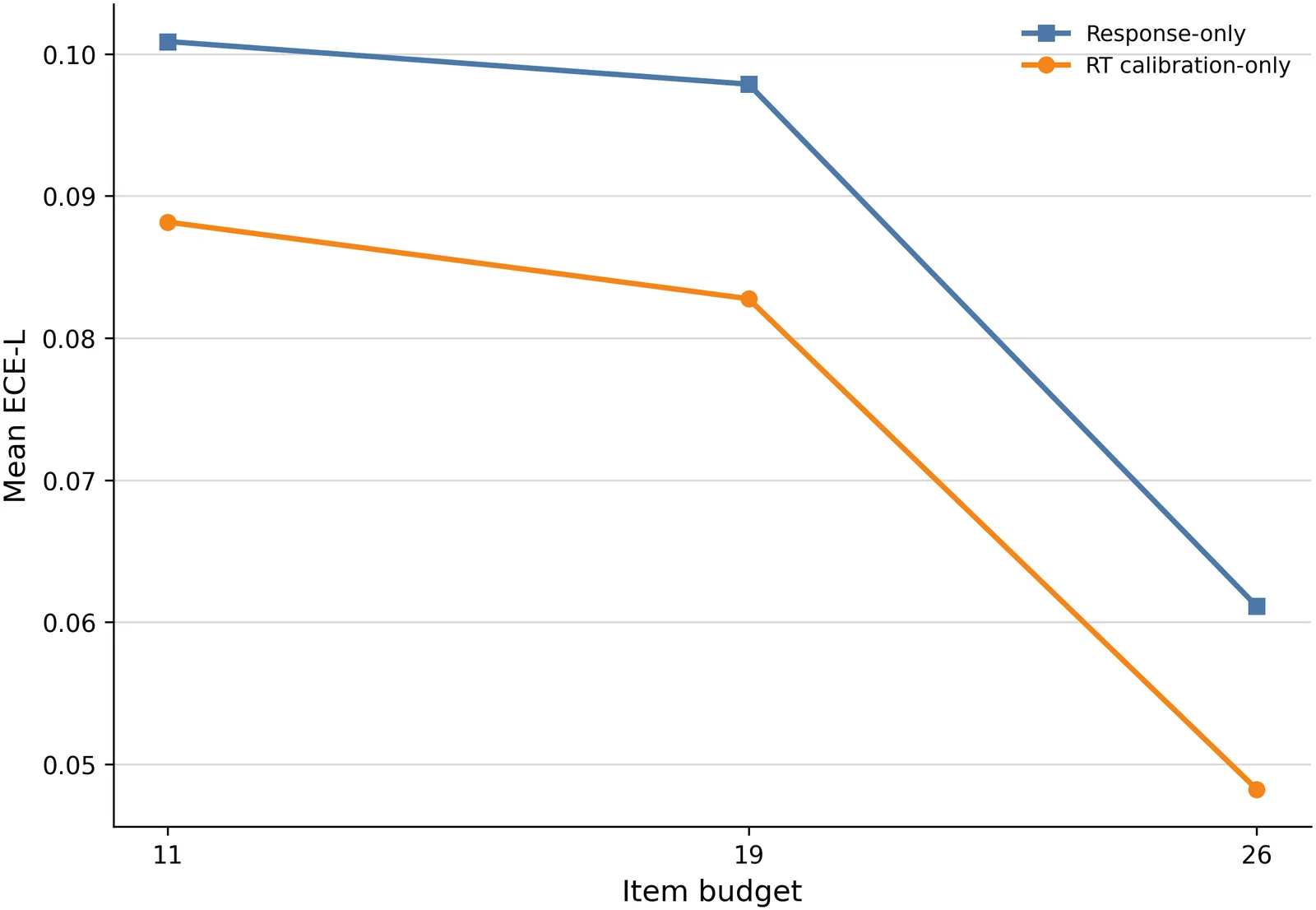
Mean label-aware expected calibration error (ECE-L) by item budget for the response-only and RT calibration-only label-preserving variants. Lower ECE-L indicates better calibration; both variants preserved the issued response-only labels.

The initial analyses tested whether RT improved score-category classification or calibration. RT did not improve classification, and standard calibration benchmarks outperformed the RT calibration-only adjustment. **Table 2** reports classification and Brier-score performance at 11-, 19-, and 26-item budgets. **Figure 1** shows macro-F1 across budgets; **Figure 2** shows comparable ECE-L values for the two label-preserving variants.

**Table 2:** Classification and Brier-score performance across reduced-item budgets.

Item budget	Variant	Macro-F1	Elevated-score recall	Brier score
11	Response-only	0.632	0.881	0.298
11	RT calibration-only	0.632	0.881	0.29
11	RT score-adjusted	0.624	0.872	0.286
19	Response-only	0.689	0.944	0.26
19	RT calibration-only	0.689	0.944	0.25
19	RT score-adjusted	0.676	0.937	0.247
26	Response-only	0.809	0.946	0.184
26	RT calibration-only	0.809	0.946	0.178
26	RT score-adjusted	0.765	0.87	0.173

Macro-F1, macro-averaged F1 score; RT, response time.

At 19 items, the response-only screen achieved a macro-F1 of 0.689 and an ECE-L of 0.098. The RT calibration-only adjustment preserved the issued labels and reduced ECE-L to 0.083. The RT score adjustment changed labels, lowered ECE-A from 0.098 to 0.079, and reduced macro-F1 from 0.689 to 0.676. Macro-F1 increased, and Brier score decreased as more items were observed (**Table 2**; **Figure 1**). ECE-L for the label-preserving variants decreased with item budget (**Figure 2**).

We compared the response-only screen with temperature scaling, one-vs-rest isotonic calibration, constant confidence softening, shuffled-RT placebo calibration, the RT calibration-only adjustment, and the RT score-adjusted variant. **Table 3** reports these 19-item calibration controls.

**Table 3:** Calibration-control comparison for the 19-item reduced screen.

Calibration method	RT information	Labels fixed	Macro-F1	Brier score	ECE-L	ECE-A	NLL
Response-only kernel baseline	No	Yes	0.689	0.26	0.098	0.098	0.796
Temperature scaling	No	Yes	0.689	0.243	0.08	0.079	0.438
One-vs-rest isotonic calibration	No	Yes	0.689	0.141	0.038	0.031	0.247
Constant confidence softening	No	Yes	0.689	0.25	0.071	0.07	0.607
Shuffled-RT placebo calibration	Shuffled	Yes	0.689	0.253	0.078	0.078	0.659
Observed RT calibration-only	Yes	Yes	0.689	0.25	0.083	0.083	0.587
RT score-adjusted	Yes	No	0.676	0.247	0.085	0.079	0.672

For label-fixed calibration controls, Macro-F1 was computed from the preserved issued labels. Brier score and NLL evaluate the full calibrated probability distribution; ECE-L evaluates the calibrated probability assigned to the preserved issued label; ECE-A evaluates calibration using the argmax label of the calibrated probability distribution. ECE-A, argmax-based expected calibration error; ECE-L, label-aware expected calibration error; Macro-F1, macro-averaged F1 score; NLL, negative log-likelihood; RT, response time.

One-vs-rest isotonic calibration achieved ECE-L = 0.038, compared with 0.083 for the RT calibration-only adjustment. The RT adjustment also did not outperform constant confidence softening; the difference in ECE-L was 0.001, with a 95% confidence interval (CI) of −0.004 to 0.007. For the label-changing RT score-adjusted variant, ECE-L was 0.085, and ECE-A was 0.079.

**Supplementary Figure 2** shows per-scale reliability diagrams, and **Supplementary Figure 3** compares ECE-L and Brier scores across calibration controls. Extending the 19-item search grid to smoothing and temperature values of 10 changed the selected PSS-14 parameters but did not change the comparative calibration conclusion. The controls did not support a general classification benefit or a calibration advantage over standard non-RT methods. The remaining analyses therefore focused on disagreement auditing.

### 3.2 Short-form disagreement burden

Short-form disagreement varied markedly across scales. **Table 4** reports scale-specific classification and Brier-score performance at 19 items. PHQ-9 and GAD-7 had higher macro-F1 than PSS-14. PSS-14 had the lowest macro-F1 and the highest Brier score.

**Table 4:** Scale-specific classification and Brier-score performance for the 19-item reduced screen.

Scale	Variant	Macro-F1	MAE	Elevated-score recall	Brier score
PHQ-9	Response-only	0.713	0.108	0.969	0.202
PHQ-9	RT calibration-only	0.713	0.108	0.969	0.204
PHQ-9	RT score-adjusted	0.713	0.104	0.954	0.188
GAD-7	Response-only	0.837	0.039	0.976	0.043
GAD-7	RT calibration-only	0.837	0.039	0.976	0.042
GAD-7	RT score-adjusted	0.804	0.038	0.964	0.043
PSS-14	Response-only	0.462	0.553	0.932	0.707
PSS-14	RT calibration-only	0.462	0.553	0.932	0.667
PSS-14	RT score-adjusted	0.466	0.543	0.934	0.675
ISI	Response-only	0.742	0.047	0.897	0.089
ISI	RT calibration-only	0.742	0.047	0.897	0.089
ISI	RT score-adjusted	0.723	0.047	0.897	0.082

MAE, mean absolute error; Macro-F1, macro-averaged F1 score; RT, response time.

Because recall alone can increase when a screen flags more participants, we also examined precision, specificity, false-positive rate, and flagged burden. **Table 5** reports these operating characteristics for the 19-item response-only screen.

**Table 5:** Elevated-score classification performance and flagged burden for the 19-item reduced screen.

Scale	Complete-questionnaire elevated-score cases	Flagged cases	Positive predictive value	Recall	Specificity	False-positive rate
PHQ-9	195	293	0.645	0.969	0.97	0.03
GAD-7	84	92	0.891	0.976	0.997	0.003
PSS-14	424	1430	0.276	0.932	0.679	0.321
ISI	39	67	0.522	0.897	0.991	0.009

Among 14,576 scale-level test decisions, the response-only 19-item screen disagreed with the complete-questionnaire category in 2,270 cases (15.6%). PSS-14 accounted for 1,563 disagreements and had a disagreement rate of 42.9%. GAD-7 and ISI had disagreement rates of 3.9% and 4.7%, respectively. ES-FN events were rare (n = 41). A total of 579 decisions (4.0%) met the pooled operational HCD definition. **Table 6** shows the distribution by scale; 492 HCD events (85.0%) occurred in PSS-14. Scale-specific threshold analyses were therefore used to examine the dependence of HCD results on the pooled threshold.

**Table 6:** Distribution of short-form disagreement, elevated-score false negatives, and high-confidence disagreements at 19 items.

Scale	Decisions	Short-form disagreements, n (%)	Elevated-score false negatives, n	High-confidence disagreements, n (%)
PHQ-9	3644	393 (10.8)	6	34 (0.9)
GAD-7	3644	142 (3.9)	2	20 (0.5)
PSS-14	3644	1,563 (42.9)	29	492 (13.5)
ISI	3644	172 (4.7)	4	33 (0.9)
All scale decisions	14576	2,270 (15.6)	41	579 (4.0)

### 3.3 Ranking short-form disagreement and HCD

RT features added little discrimination for all short-form disagreements. AP was 0.488 with confidence plus RT, 0.467 with confidence alone, and 0.438 with confidence plus shuffled RT (**Table 7**). For HCD, the corresponding values were 0.772, 0.540, and 0.491; ROC-AUC for the shuffled-RT model was 0.918. Within the fixed high-confidence gate, AP was 0.739, 0.628, and 0.561. Within the fixed high-confidence gate, the AP difference between the confidence-plus-RT and confidence-only models was 0.111 (95% CI, 0.093 to 0.131). **Supplementary Tables 5**–**S7** report threshold sensitivity and bootstrap results.

**Table 7:** Ranking short-form disagreement and high-confidence disagreement using confidence and response-time predictor sets.

Outcome	Predictor set	AP	ROC-AUC
Short-form disagreement (n = 2,270)	Confidence only	0.467	0.863
Short-form disagreement (n = 2,270)	Response time only	0.409	0.812
Short-form disagreement (n = 2,270)	Confidence + response time	0.488	0.868
Short-form disagreement (n = 2,270)	Confidence + shuffled response time	0.438	0.86
High-confidence disagreement (n = 579)	Confidence only	0.54	0.952
High-confidence disagreement (n = 579)	Response time only	0.557	0.886
High-confidence disagreement (n = 579)	Confidence + response time	0.772	0.958
High-confidence disagreement (n = 579)	Confidence + shuffled response time	0.491	0.918

AP, average precision; ROC-AUC, area under the receiver operating characteristic curve.

### 3.4 Selective escalation to questionnaire completion

**Figure 3:**
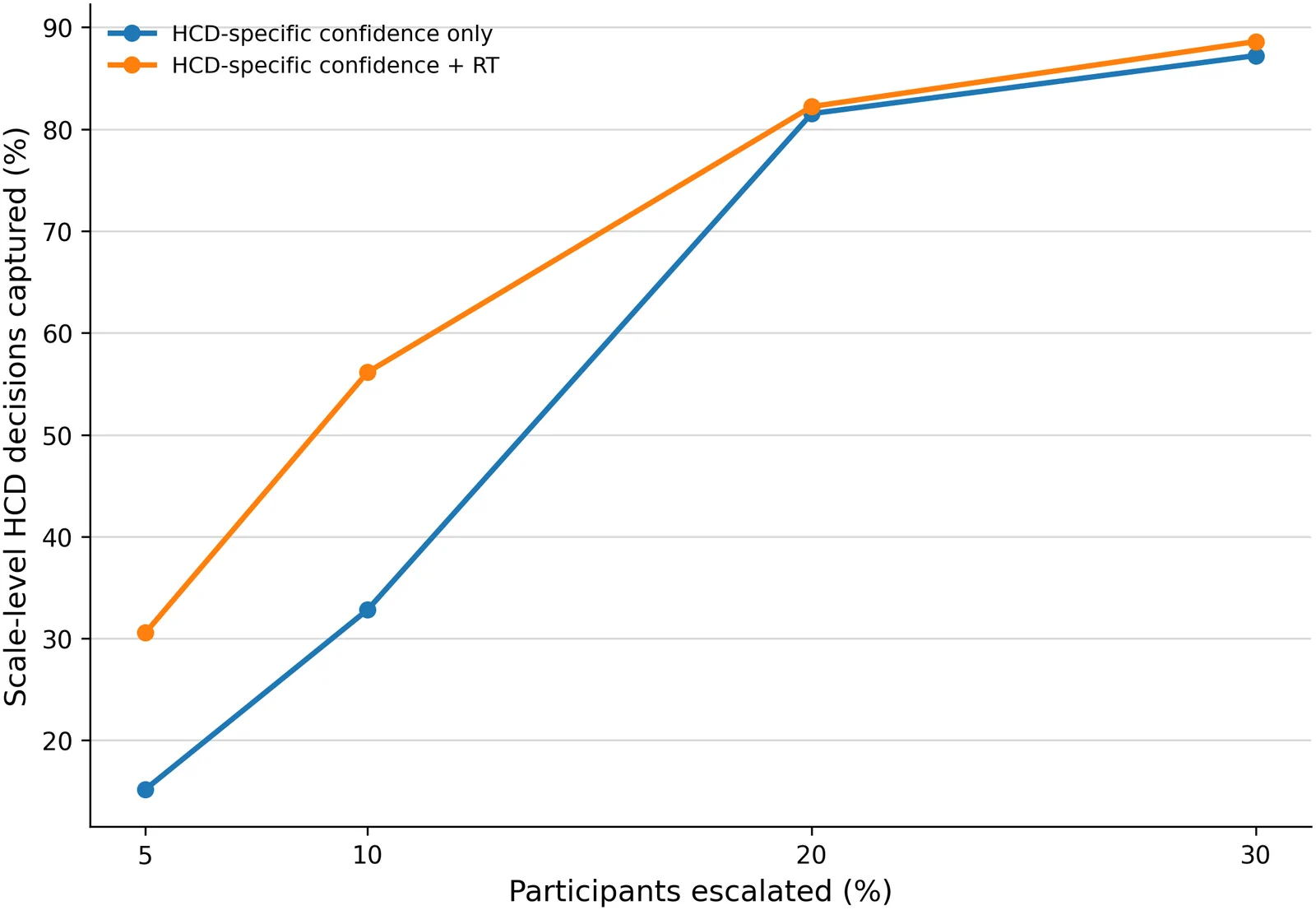
Scale-level HCD capture across participant-level escalation capacities. Curves compare the HCD-specific confidence-only model with the otherwise identical confidence-plus-RT model at 5%, 10%, 20%, and 30% participant-escalation rates.

The analysis tested whether participant-level escalation rankings improved capture of scale-level HCD decisions. **Figure 3** and **Table 8** report the target-matched capacity results across 5%, 10%, 20%, and 30% participant-escalation rates. When 5% of participants were escalated, 30.6% of the 579 scale-level HCD decisions were captured, compared with 15.2% for confidence alone. At 10% participant escalation, the corresponding values were 56.1% and 32.8%. The differences were 15.4 percentage points (95% CI, 11.9 to 18.7) and 23.3 points (95% CI, 18.9 to 27.8), respectively. Differences at 20% and 30% were 0.7 and 1.4 points, and both confidence intervals included zero. **Supplementary Table S8** provides a descriptive multi-strategy comparison at the 20% operating point. At this operating point, the RT-only ranking captured 63.7% of scale-level HCD decisions, compared with 20.2% under random selection. The target-matched confidence-plus-RT model captured 82.2%, compared with 81.5% for confidence alone. **Supplementary Tables 9**–**S12** report bootstrap uncertainty and scale-specific results. ES-FN results are descriptive because only 41 events occurred.

**Table 8:** Target-matched scale-level HCD capture across participant-level escalation capacities.

Escalation capacity (%)	Confidence only (%)	Confidence + RT (%)	Difference (percentage points)	95% CI
5	15.2	30.6	15.4	11.9 to 18.7
10	32.8	56.1	23.3	18.9 to 27.8
20	81.5	82.2	0.7	-1.7 to 3.5
30	87.2	88.6	1.4	-0.3 to 2.7

HCD, high-confidence disagreement; CI, participant-cluster bootstrap confidence interval. Escalation capacity denotes the percentage of test participants selected for completion of the remaining questionnaire items.

**Figure 3** shows scale-level HCD capture across participant-level escalation capacities. **Supplementary Figure 4** shows the overall disagreement-capture frontier. The largest RT-related gains occurred at the 5% and 10% escalation capacities. At 20% and 30% capacity, the differences were small, and their confidence intervals included zero.

### 3.5 Process-quality strata

**Figure 4:**
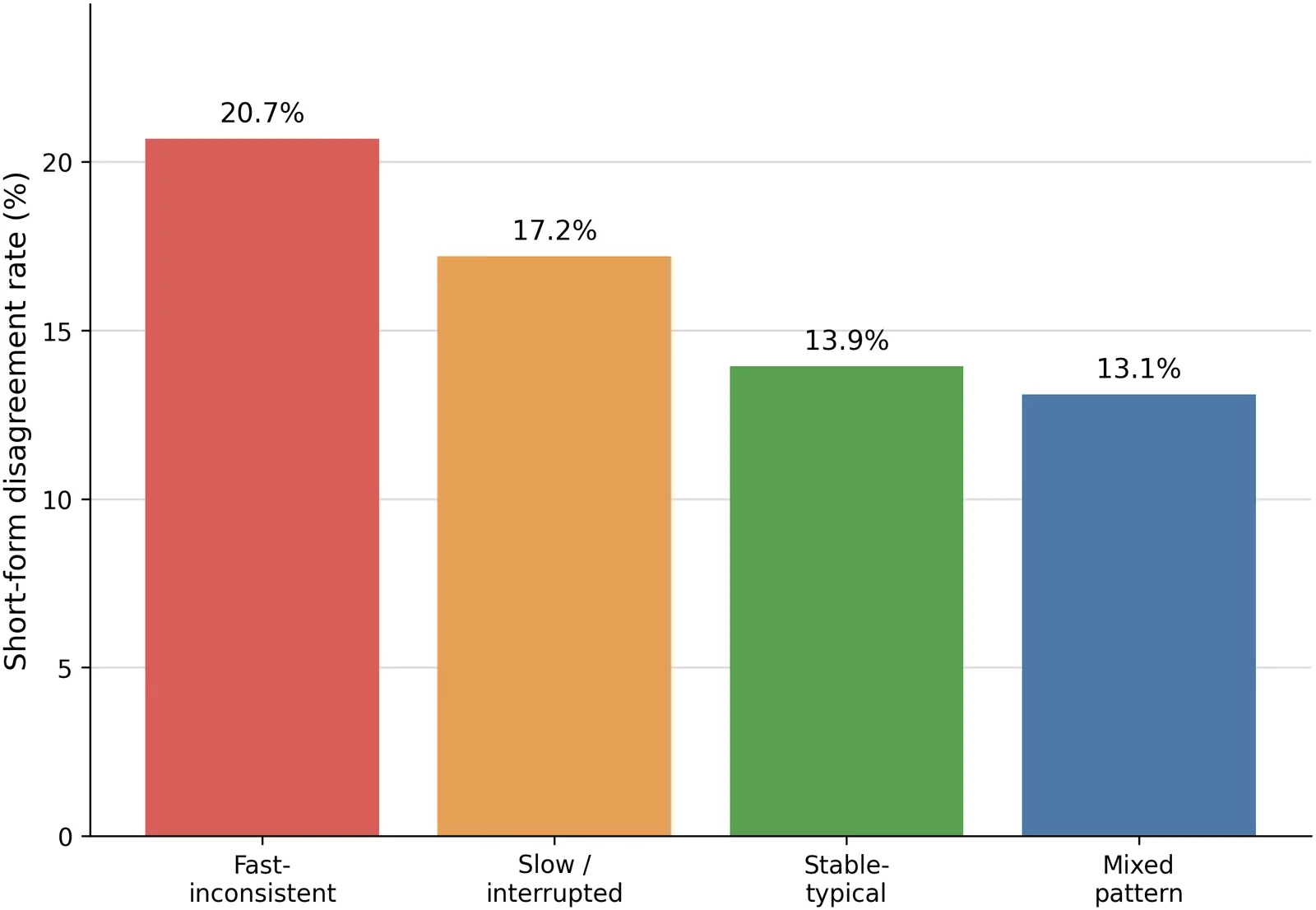
Short-form disagreement rates across RT process-quality strata derived from the response-only 19-item selected-item set.

**Table 9** summarizes disagreement, HCD, calibration, and process consistency across selected-item process-quality strata; **Figure 4** shows disagreement rates.

**Table 9:** Short-form decision quality across selected-item process-quality strata.

Process-quality stratum	Disagreement (%)	HCD (%)	ECE-L	Mean process-consistency score
Fast-inconsistent (n = 318)	20.7	10.1	0.154	0.765
Slow/interrupted (n = 1,506)	17.2	4.4	0.089	0.845
Stable-typical (n = 513)	13.9	1.1	0.048	0.95
Mixed pattern (n = 1,307)	13.1	3.1	0.071	0.874

Disagreement, short-form disagreement with the complete-questionnaire category; HCD, high-confidence disagreement; the mean process-consistency score is the operational selected-item response-process index defined in Equation 14, not a correlation or reliability coefficient.

The fast-inconsistent stratum had the highest disagreement rate (20.7%), HCD rate (10.1%), and ECE-L (0.154), although it included only 318 participants. The shuffled-RT control did not show the same concentration of disagreement and HCD in the fast-inconsistent stratum. Per-scale analyses showed that the pooled HCD result was concentrated in PSS-14.

### 3.6 Robustness, baselines, leakage, and transfer

In the response-rich sensitivity analysis, adding RT increased AP for HCD ranking by 0.007 (95% CI, -0.001 to 0.017) and did not improve ordinary-disagreement ranking (**Supplementary Table 13**).

The short-form-seeded fixed subset had higher macro-F1 than both adaptive policies, and repeated random subsets remained competitive (**Table 10**). The mask-aware CART and logistic models provided lightweight response-only comparators. Strict observed-first-19, near-balanced fixed-19, and participant-specific adaptive replay results are reported in **Supplementary Table 14**.

**Table 10:** Performance of fixed, random, adaptive, and learned reduced-item policies.

Reduced-item policy	Macro-F1	ECE-L	Elevated-score recall	Brier score
Fixed-order truncation	0.662	0.124	0.565	0.271
Short-form-seeded fixed subset	0.751	0.067	0.837	0.23
Training-selected fixed subset	0.706	0.082	0.905	0.249
Repeated random subsets (50)	0.717	0.025	0.772	0.182
Response-only adaptive selection	0.689	0.098	0.944	0.26
RT score-adjusted adaptive policy	0.676	0.085	0.937	0.247
Training-selected CART	0.604	0.072	0.87	0.239
Training-selected logistic regression	0.498	0.086	0.295	0.175
Short-form-seeded CART	0.633	0.069	0.834	0.231
Short-form-seeded logistic regression	0.498	0.09	0.294	0.181

CART, classification and regression tree; ECE-L, label-aware expected calibration error; Macro-F1, macro-averaged F1 score; RT, response time. Logistic regression models used default probability-to-class conversion without additional threshold tuning.

Across nine utility specifications, macro-F1 ranged from 0.686 to 0.698 and ECE-L from 0.095 to 0.099. At 20% escalation, any-disagreement capture ranged from 25.6% to 29.8%, and HCD capture ranged from 76.1% to 82.5% (**Supplementary Table 4**). Classification and calibration were stable across specifications, whereas audit ranking changed more when coverage-related terms were removed.

Scale-specific validation thresholds reduced PSS-14 dominance in the HCD target. Under the inclusive rule, 709 test HCD events were identified, of which 55.0% were from PSS-14. Under the strict rule, 463 events remained, and 61.6% were from PSS-14; the pooled primary threshold had assigned 85.0% of HCD events to PSS-14. The RT increment remained largest and most stable for PSS-14. PHQ-9 showed a positive increment under the inclusive rule, but the estimate was smaller and uncertain after threshold ties were excluded. GAD-7 and ISI did not show stable increments. These results do not support a common cross-scale RT rule. Full results are reported in **Supplementary Table 15**.

The content residualization model explained little RT variation (R² = 0.038; **Supplementary Table 2**). Consistent with recommendations for leakage control and reproducibility in machine-learning research (45), all reported checks passed, including split integrity, training-only residualization, and fixed-prefix invariance (**Supplementary Table 16**). The response-speed subgroup analysis used RT summaries from the selected 19-item set and remained exploratory because it did not include formal inferential testing (**Supplementary Figure 5**).

Leave-one-scale-out experiments tested cross-scale transfer of ordinary-disagreement ranking models. When trained on the other three scales, the confidence-plus-RT model underperformed the confidence-only model for every held-out scale (**Supplementary Table 17**). These ordinary-disagreement models did not transfer across held-out scales. Cross-scale transfer of HCD ranking was not directly evaluated.

Complete-questionnaire item responses had Cronbach’s α values of 0.881 for PHQ-9, 0.903 for GAD-7, 0.793 for PSS-14, and 0.865 for ISI (**Supplementary Table 18**). These values provide a descriptive data-quality check; they do not establish diagnostic validity or unidimensionality.

A score-level sensitivity analysis reported cutoff-free full-score MAE and, separately, near-boundary disagreement based on the operational category boundaries (**Supplementary Table 19**). PSS-14 had a full-score MAE of 8.249, compared with 0.888 for PHQ-9, 0.330 for GAD-7, and 0.965 for ISI. In the aggregate excluding PSS-14, full-score MAE was 0.728, and 15.8% of near-boundary decisions disagreed across the reduced and complete scores. These results indicate that pooled category-level findings were heavily influenced by PSS-14.

## 4 Discussion

RT improved HCD ranking at the 5% and 10% escalation rates, increasing capture by 15.4 and 23.3 percentage points over confidence alone. RT did not materially improve overall score-category classification. Although the RT calibration-only adjustment reduced ECE-L relative to the response-only kernel baseline, standard non-RT calibration methods performed better. The HCD gain diminished after detailed response features were added.

PSS-14 contributed 1,563 of 2,270 disagreements and 492 of 579 HCD events, and its score-approximation error was substantially larger than that of the other scales. Published evidence indicates version- and study-specific variation in the measurement structure of the PSS-10 and PSS-14 (35). The pooled HCD result was therefore scale-dependent. The 41 ES-FN events were insufficient for a stable estimate of RT performance on missed elevated-score decisions.

Previous research has used RT to characterize heterogeneous response states, careless responding, and cognitive processing speed (13, 14, 20). Such applications do not justify treating RT as a direct index of symptom severity or response validity. In the present study, RT was therefore interpreted as a response-process signal rather than as a measure of these constructs. The shuffled-RT control did not reproduce the HCD-ranking increment observed with RT, suggesting that the gain did not arise solely from the addition of predictor dimensions. The AP increment fell to 0.007 in the response-rich models, suggesting that some of the information provided by RT overlapped with information already contained in the detailed selected-response patterns.

Selective prediction treats deferral as a trade-off between coverage and disagreement among accepted decisions (21–24). Consistent with this framework, the incremental value of RT was greatest when escalation capacity was lowest. At 20% and 30%, the confidence-only model had already captured most HCD events, leaving little room for additional RT information. This pattern indicates that the contribution of RT lies primarily in prioritizing participants when questionnaire-completion capacity is most constrained.

Research on reduced-item mental health assessment commonly evaluates how many items can be removed while preserving scores or classification (2, 6–9). Building on this work, the present study examined how to decide which provisional reduced-item results should trigger completion of the remaining items. The simulated workflow used a common 19-item budget with participant-specific item selection, followed by selective completion of the remaining items, so the total simulated questionnaire length was either 19 or 37 items. The short-form-seeded fixed subset and repeated random subsets remained competitive, indicating that the main contribution of the proposed workflow lies in selective completion rather than in identifying a single universally optimal item-selection rule. The item-priority score emphasized empirical associations with complete-questionnaire categories and response variability, consistent with the operational objective of approximating complete-questionnaire categories from existing rule-scored instruments. Discrepancies between reduced-item and complete-questionnaire categories may reflect a combination of the empirical item-selection procedure, score proration, limited item coverage, and proximity to category boundaries. The practical contribution of the workflow lies in adding a capacity-constrained completion stage to existing rule-scored digital questionnaires without requiring the prior construction of a calibrated item bank. At 5% and 10% escalation, the simulated mean questionnaire burden was 19.9 and 20.8 items per participant, respectively, compared with 37 items under universal completion. Classification and calibration were broadly stable across RT residualization choices and utility perturbations, although audit capture changed when coverage-related terms were removed. Scale-specific thresholds reduced PSS-14 dominance, and leave-one-scale-out models did not support cross-scale transfer for ordinary-disagreement ranking.

From a public mental health perspective, selective completion could reduce questionnaire burden for participants not selected for escalation while concentrating limited full-questionnaire completion capacity on provisional results with the highest estimated risk of HCD. The workflow can be adapted to other digital questionnaire settings by adjusting the initial item budget, scoring model, escalation threshold, and completion capacity to the instrument and service constraints. Its core inputs can be captured or derived in computerized assessments, supporting potential applications in student screening, clinical intake, and repeated symptom monitoring.

Several limitations constrain interpretation. The observed responses and RTs were generated under the original fixed administration order and may differ from those produced during prospective adaptive administration. The HCD event set depended on the response-only selection and prorated scoring pipeline, and alternative non-RT baselines could change the distribution of disagreements, particularly for PSS-14. HCD was partly defined by confidence; the audit models included confidence-derived predictors, a pooled threshold was applied across scales, and the same validation split was used for threshold definition and hyperparameter selection. The sample comprised a single online student cohort, and the reference outcomes were questionnaire score categories rather than clinical diagnoses. Bootstrap intervals were conditional on the fitted procedure. Subgroup and sensitivity analyses were exploratory and were not adjusted for multiple comparisons.

Future studies should collect RT under the intended administration design, adopt robust non-RT and scale-specific baselines, and separate threshold derivation from model tuning. Independent external validation should assess the workflow in clinically characterized samples and prospective testing environments. Such evaluations should examine participant burden, completion behavior, operational feasibility, and the incremental value of RT beyond the response features already available in deployed screening systems. Where the workflow is used to support clinical screening, its effects on referral and downstream assessment decisions should also be evaluated.

## 5 Conclusion

Response-time signals improved prioritization within a selective-escalation workflow when full-questionnaire completion capacity was most constrained. At 5% and 10% escalation, adding RT increased HCD capture by 15.4 and 23.3 percentage points over confidence alone, while overall score-category classification did not materially improve. These findings support RT as a complementary operational signal for allocating limited full-questionnaire completion capacity. In large-scale mental health screening, the workflow could reduce average questionnaire burden while directing additional assessment toward provisional results with the highest estimated risk of HCD. The observed benefit was strongest for PSS-14 and warrants prospective evaluation across scales, independent samples, and live screening settings.

## Data Availability

Publicly available datasets were analyzed in this study. The dataset analyzed in this study is publicly available in Zenodo: Su, Z. (2023), “Temporal dynamics in psychological assessments: a novel dataset with scales and response times,” Version v2. Repository: Zenodo. Accession number/DOI: 10.5281/zenodo.10423537. Direct link: https://doi.org/10.5281/zenodo.10423537. An associated Zenodo record for the present study is available at https://doi.org/10.5281/zenodo.21811028 (46).
