# Supplementary material for: Response-time process signals for selective escalation in reduced-item mental health symptom screening
Source: Front Psychiatry. 2026 Aug 25;17:1926874. doi: 10.3389/fpsyt.2026.1926874 (PMC13550221; doi:10.3389/fpsyt.2026.1926874)
Supplement: Supplementary file 1 [file SupplementaryFile1.docx]

Supplementary Material

# 1 Supplementary Tables

**Supplementary Table S1.** Complete offline workflow for reduced-item selection, calibration, decision auditing, and selective escalation.

| **Step** | **Stage** | **Operation and information boundary** |
| --- | --- | --- |
| 1 | RT preprocessing | Estimate item-specific 0.5th and 99.5th percentile RT winsorization thresholds using training participants only, and apply them unchanged to validation and test participants. |
| 2 | RT residualization | Fit both ordinary least squares residualization specifications using training item records only, and standardize residuals using training-derived moments. |
| 3 | Item-priority scoring | Compute the supervised item-priority score $I_{j}$ using training participants only; initialize each scale with the item having the highest $I_{j}$. |
| 4 | Selected-item state update | Update selected-item scores, boundary distances, coverage, uncertainty, and, for the RT-aware policy, process consistency using selected and answered items only. |
| 5 | Candidate ranking | Calculate the utility of each remaining item from the training-derived item-priority scores and the current selected-item state; no unselected response or RT is accessed. |
| 6 | Sequential selection | Select the maximum-utility item, resolve ties deterministically without response or RT information, and repeat Steps 4-6 until the budget is reached. |
| 7 | Score-category prediction | At the final budget, calculate prorated scores from selected responses and map them to dataset-defined score categories. RT does not enter the scoring equation directly, although it may affect labels through item selection. |
| 8 | Probability calibration | Select scale-specific smoothing parameters by validation expected calibration error (ECE) and apply them unchanged to the test set. In the RT calibration-only control, multiply $\sigma_{s}$ by $1+1.25(1-R_{\mathrm{pB}})$, where $R_{\mathrm{pB}}$ is the participant-level consistency score at final budget $B$; the response-only label remains unchanged. |
| 9 | Audit features | Construct confidence, entropy, probability-margin, boundary-distance, coverage, and selected-item RT features. Complete-questionnaire labels are used only to define supervised audit targets and evaluation outcomes. |
| 10 | Audit models | Fit class-balanced L2 logistic audit models for short-form disagreement, operational HCD, and elevated-score false negatives. Select C by validation average precision (AP); refit with the seed-fixed audit-training sample of up to 5,000 participants and all validation participants; then evaluate once on the complete test split. |
| 11 | Escalation ranking | Define participant-level risk as the maximum of the four scale-level audit scores and rank participants accordingly. No universal raw-RT cutoff directly triggers escalation. |
| 12 | Completion and evaluation | At each escalation capacity, simulate completion of the remaining items by the highest-risk participants and report burden, target capture, and post-escalation agreement. |

**Note:** Selection variables, prediction features, and audit predictors used only information from items selected and answered at the relevant stage. Complete-questionnaire responses and score-derived labels were used to define supervised audit targets, simulate completion outcomes, and evaluate performance. Escalation percentages represent resource-capacity scenarios and are not clinically validated decision thresholds.

**Supplementary Table S2.** Sensitivity of response-time residualization to alternative preprocessing models.

| **Residual model** | **Training R²** | **Residual SD** | **5th percentile** | **Median** | **95th percentile** |
| --- | --- | --- | --- | --- | --- |
| Content model, 0.5-99.5% winsorization | 0.038 | 0.647 | -1.593 | -0.027 | 1.655 |
| Item fixed-effect model, 0.5-99.5% winsorization | 0.172 | 0.600 | -1.619 | -0.039 | 1.660 |
| Scale-specific content model, 0.5-99.5% winsorization | 0.086 | 0.630 | -1.607 | -0.029 | 1.655 |
| Content model, 1-99% winsorization | 0.039 | 0.636 | -1.615 | -0.023 | 1.688 |

**Note:** For the two residualization specifications reported in the manuscript, the primary content-adjusted model had R² = 0.038 and residual SD = 0.647, and the response-conditioned sensitivity model had R² = 0.057 and residual SD = 0.641. The other rows evaluate alternative residual specifications and winsorization limits.

**Supplementary Table S3.** Fixed operational coefficients for item-priority scoring, response-process consistency, item-selection utility, and label-preserving probability calibration.

| **Component** | **Fixed value** | **Operational role** | **Provenance and data boundary** | **Sensitivity treatment in Supplementary Table S4** |
| --- | --- | --- | --- | --- |
| Process-consistency score | Fast $z<-1$: 0.35; long $z>1$: 0.20; residual SD: 0.08; SD cap 4; lower bound 0.10 | Summarizes RT inconsistency among selected and answered items | Specification fixed during model development; selected-item RT residuals only | RT scaling and removal of the uncertainty channel |
| Response-variability term | 0.10 | Adds uncertainty when selected responses within a scale are heterogeneous | Coefficient fixed during model development | Held fixed |
| RT uncertainty inflation | $0.25(1-R_{pt})$ | Inflates scale uncertainty when process consistency is lower | Coefficient fixed during model development | $m=0,0.5,1,2$  remove increment |
| Coverage weight | $0.20+0.40(1-R_{pt})$ | Encourages representation of incompletely covered scales | Base value and RT increment fixed during model development | $m=0,0.5,1,2$  remove term |
| Under-covered bonus | 0.08 | Favors scales tied for the fewest selected items | Coefficient fixed during model development | Removal of the term |
| Redundancy penalty | $0.015+0.020(1-R_{pt})$ | Discourages repeated selection from one scale | Base value and RT increment fixed during model development | $m=0,0.5,1,2$  remove term |
| Confirmation weight | $0.25(1-R_{pt})$ | Favors confirmation using higher-priority items when consistency is lower | Coefficient fixed during model development | $m=0,0.5,1,2$  remove term |
| Variance weight in the item-priority score | $\text{ω}_{\text{I}}\text{= 0.05}$ | Adds a small training-set response-variance contribution to the item-priority score | Item statistics estimated using training participants only; no test-set optimization. | Held fixed |
| RT calibration-only unreliability weight | $\text{γ}_{\text{RT}}\text{= 1.25}$ | Expands participant-specific probability smoothing as process consistency decreases | Scale-specific smoothing selected on validation data; no test-set optimization | Held fixed |

**Note:** These operational coefficients specify the algorithmic implementation evaluated in the replay analyses. They were fixed during model development, and test outcomes were used only to evaluate the fixed rule and the post hoc sensitivity variants. They should be interpreted as algorithmic settings, with no standalone psychometric or clinical meaning. The constants $\text{ω}_{\text{I}}\text{= 0.05}$ and $\text{γ}_{\text{RT}}\text{= 1.25}$ were already implemented in the analysis code and were held fixed across all variants in Supplementary Table S4.

**Supplementary Table S4. Sensitivity of the 19-item RT-aware item-selection utility to fixed operational coefficients.**

| **Variant** | $\boldsymbol{m}$ **/ ablation** | **Utility change** | **Macro-F1 / ECE-L** | **Disagreement / HCD, n** | **20% disagreement / HCD capture, %** |
| --- | --- | --- | --- | --- | --- |
| No RT modulation | 0.0 | Set all RT-dependent increments to zero | 0.689 / 0.098 | 2,270 / 579 | 27.0 / 82.2 |
| Half RT modulation | 0.5 | Multiply all RT-dependent increments by 0.5 | 0.691 / 0.098 | 2,254 / 578 | 26.8 / 82.2 |
| Reference RT-aware utility | 1.0 | Coefficients fixed during model development and retained unchanged for test-set evaluation | 0.688 / 0.099 | 2,252 / 598 | 26.5 / 76.6 |
| Double RT modulation | 2.0 | Multiply all RT-dependent increments by 2 | 0.686 / 0.096 | 2,262 / 594 | 25.6 / 76.1 |
| No RT uncertainty inflation | 1.0 | Remove the RT increment in score uncertainty | 0.688 / 0.099 | 2,255 / 595 | 26.3 / 76.5 |
| No coverage term | 1.0 | Remove $\lambda_{pt}C_{pst}$ | 0.696 / 0.095 | 2,208 / 554 | 29.8 / 82.5 |
| No under-covered bonus | 1.0 | Remove $0.08A_{pst}$ | 0.698 / 0.095 | 2,266 / 576 | 28.1 / 81.8 |
| No redundancy penalty | 1.0 | Remove $-\rho_{pt}N_{pst}$ | 0.689 / 0.096 | 2,253 / 566 | 27.1 / 82.5 |
| No confirmation term | 1.0 | Remove $\eta_{pt}I_{j}(1+A_{pst})$ | 0.691 / 0.097 | 2,256 / 575 | 26.7 / 81.7 |

**Note:** The multiplier m scales only the RT-dependent increments. All variants retained the original participant splits, training-derived item-priority scores, response-only prorated score-category assignment, and validation-selected scale-specific probability smoothing. For each variant, the HCD threshold was re-estimated from validation-set disagreements, and the target-specific confidence-plus-RT audit models for disagreement and HCD were refitted. Because the variants could select different 19-item subsets, their disagreement and HCD event counts may differ from those in the main response-only audit frame. Displayed values are rounded; machine-readable CSV files contain full-precision results.

**Supplementary Table S5.** Scale-decision-level sensitivity of HCD ranking to the validation confidence-threshold quantile.

| **Validation quantile** | **Model** | **Test HCD, n** | **AP** | **ROC-AUC** | **Recall at the top 10%** | **Precision at the top 10%** |
| --- | --- | --- | --- | --- | --- | --- |
| 0.70 | Confidence only | 712 | 0.297 | 0.893 | 0.692 | 0.338 |
| 0.70 | RT only | 712 | 0.499 | 0.852 | 0.659 | 0.322 |
| 0.70 | Confidence + RT | 712 | 0.614 | 0.952 | 0.770 | 0.376 |
| 0.70 | Confidence + shuffled RT | 712 | 0.368 | 0.933 | 0.744 | 0.364 |
| 0.75 | Confidence only | 579 | 0.540 | 0.952 | 0.850 | 0.337 |
| 0.75 | RT only | 579 | 0.557 | 0.886 | 0.770 | 0.306 |
| 0.75 | Confidence + RT | 579 | 0.772 | 0.958 | 0.858 | 0.341 |
| 0.75 | Confidence + shuffled RT | 579 | 0.491 | 0.918 | 0.850 | 0.337 |
| 0.80 | Confidence only | 453 | 0.551 | 0.959 | 0.938 | 0.291 |
| 0.80 | RT only | 453 | 0.556 | 0.936 | 0.881 | 0.274 |
| 0.80 | Confidence + RT | 453 | 0.821 | 0.986 | 0.962 | 0.299 |
| 0.80 | Confidence + shuffled RT | 453 | 0.479 | 0.961 | 0.934 | 0.290 |
| 0.90 | Confidence only | 291 | 0.580 | 0.978 | 0.976 | 0.195 |
| 0.90 | RT only | 291 | 0.412 | 0.956 | 0.938 | 0.187 |
| 0.90 | Confidence + RT | 291 | 0.778 | 0.986 | 0.969 | 0.193 |
| 0.90 | Confidence + shuffled RT | 291 | 0.402 | 0.979 | 0.973 | 0.194 |

**Note:** Thresholds were estimated from validation-set disagreements and then applied unchanged to the test set.

**Supplementary Table S6.** Participant-bootstrap uncertainty for average-precision differences between confidence-plus-RT and comparator models.

| **Outcome** | **Comparison** | **ΔAP** | **95% CI lower** | **95% CI upper** | **Bootstrap repeats** |
| --- | --- | --- | --- | --- | --- |
| Short-form disagreement | Confidence + RT minus confidence only | 0.022 | 0.007 | 0.037 | 500 |
| Short-form disagreement | Confidence + RT minus shuffled RT | 0.050 | 0.036 | 0.064 | 500 |
| HCD | Confidence + RT minus confidence only | 0.232 | 0.204 | 0.262 | 500 |
| HCD | Confidence + RT minus shuffled RT | 0.280 | 0.239 | 0.315 | 500 |

**Note:** Bootstrap samples resampled participants with replacement and retained all scale decisions for each sampled participant. HCD = high-confidence disagreement; CI = percentile bootstrap confidence interval.

**Supplementary Table S7.** Disagreement-versus-agreement detection within the fixed high-confidence gate.

| **Model** | **Capacity (%)** | **AP** | **Delta AP vs confidence only** | **95% CI** | **Disagreements captured (%)** | **Gated disagreements, n** |
| --- | --- | --- | --- | --- | --- | --- |
| Confidence only | 5 / 10 / 20 / 30 | 0.628 | 0.000 | 0.000 to 0.000 | 15.0 / 37.1 / 85.8 / 94.6 | 579 |
| Confidence + RT | 5 / 10 / 20 / 30 | 0.739 | 0.111 | 0.093 to 0.131 | 27.1 / 50.6 / 82.7 / 94.1 | 579 |
| Confidence + shuffled RT | 5 / 10 / 20 / 30 | 0.561 | -0.066 | -0.078 to -0.054 | 14.9 / 36.4 / 76.5 / 95.0 | 579 |

**Note:** The same validation-derived high-confidence gate was applied to training, validation, and test rows before the disagreement-versus-agreement models were fitted. Higher AP did not imply higher capture at every participant capacity.

**Supplementary Table S8.** Descriptive multi-strategy comparison at the 20% participant-level escalation operating point.

| **Escalation ranking** | **All short-form disagreements captured (%)** | **HCD captured (%)** | **Elevated-score false negatives captured (%)** |
| --- | --- | --- | --- |
| Random selection | 20.0 | 20.2 | 19.2 |
| Lowest confidence | 24.5 | 5.9 | 53.7 |
| RT process score | 28.3 | 63.7 | 29.3 |
| Confidence + RT disagreement model | 27.0 | 5.9 | 24.4 |
| Confidence-only HCD model | 22.9 | 81.5 | 7.3 |
| Confidence + RT HCD model | 23.1 | 82.2 | 4.9 |
| Confidence + RT ES-FN model | 28.6 | 7.9 | 53.7 |
| Confidence + shuffled RT | 26.8 | 5.7 | 22.0 |
| Oracle | 43.3 | 100.0 | 100.0 |

**Note:** Point estimates are shown at 20% capacity. The primary incremental RT comparison is the target-matched capacity analysis in Figure 3 and Table 8 of the main manuscript; participant-bootstrap CIs for selected strategies are reported in Supplementary Table S9. RT = response time; HCD = high-confidence disagreement; ES-FN = elevated-score false negative.

**Supplementary Table S9.** Participant-bootstrap uncertainty for disagreement, HCD, and ES-FN capture at 20% selective escalation to questionnaire completion.

| **Strategy** | **Disagreement capture % (95% CI)** | **HCD capture % (95% CI)** | **ES-FN capture % (95% CI)** |
| --- | --- | --- | --- |
| Random selection | 20.0 (18.6-21.5) | 20.2 (16.8-22.8) | 19.2 (9.1-33.3) |
| Lowest confidence | 24.5 (23.1-26.3) | 5.9 (4.0-7.8) | 53.7 (39.8-70.4) |
| RT process score | 28.3 (26.9-29.4) | 63.7 (60.1-67.1) | 29.3 (13.0-43.4) |
| Confidence + RT disagreement model | 27.0 (25.6-28.3) | 5.9 (3.9-8.0) | 24.4 (11.9-41.2) |
| Confidence-only HCD model | 22.9 (21.5-24.5) | 81.5 (78.0-84.9) | 7.3 (0.0-17.4) |
| Confidence + RT HCD model | 23.1 (21.5-24.4) | 82.2 (79.1-84.8) | 4.9 (0.0-13.6) |

**Note:** Confidence intervals used participant-level bootstrap resampling with repeated participant draws retained. HCD = high-confidence disagreement; ES-FN = elevated-score false negative.

**Supplementary Table S10.** Scale-specific disagreement and HCD capture at 20% selective escalation to questionnaire completion.

| **Scale** | **Strategy** | **Disagreements, n** | **HCD, n** | **Disagreement capture %** | **HCD capture %** |
| --- | --- | --- | --- | --- | --- |
| PHQ-9 | Lowest confidence | 393 | 34 | 28.2 | 0.0 |
| PHQ-9 | RT process score | 393 | 34 | 12.0 | 14.7 |
| PHQ-9 | Confidence + RT HCD model | 393 | 34 | 8.9 | 50.0 |
| GAD-7 | Lowest confidence | 142 | 20 | 95.1 | 65.0 |
| GAD-7 | RT process score | 142 | 20 | 7.7 | 15.0 |
| GAD-7 | Confidence + RT HCD model | 142 | 20 | 9.9 | 70.0 |
| PSS | Lowest confidence | 1563 | 492 | 23.9 | 0.0 |
| PSS | RT process score | 1563 | 492 | 35.8 | 72.0 |
| PSS | Confidence + RT HCD model | 1563 | 492 | 29.8 | 94.3 |
| ISI | Lowest confidence | 172 | 33 | 82.6 | 9.1 |
| ISI | RT process score | 172 | 33 | 15.1 | 21.2 |
| ISI | Confidence + RT HCD model | 172 | 33 | 14.0 | 60.6 |

**Note:** The pooled HCD gain was driven mainly by PSS.

**Supplementary Table S11.** Disagreement and HCD capture at 20% participant escalation after excluding PSS.

| **Strategy** | **Disagreements, n** | **HCD, n** | **Disagreement capture %** | **HCD capture %** |
| --- | --- | --- | --- | --- |
| Random selection | 707 | 87 | 20.1 | 20.1 |
| Lowest confidence | 707 | 87 | 36.6 | 23.0 |
| RT process score | 707 | 87 | 11.9 | 17.2 |
| Confidence + RT HCD model | 707 | 87 | 32.2 | 77.0 |

**Note:** The same 20% participant-level escalation budget was applied to PHQ-9, GAD-7, and ISI decisions. The supervised HCD ranking remained informative, whereas the unsupervised RT-only score did not show a general advantage.

**Supplementary Table S12.** Scale-specific and non-PSS performance of target-matched HCD ranking models.

| **Analysis set** | **HCD n** | **Conf.-only AP** | **Conf.+RT AP** | **Delta AP (95% CI)** | **5% capture C / C+RT** | **10% capture C / C+RT** | **20% capture C / C+RT** |
| --- | --- | --- | --- | --- | --- | --- | --- |
| PHQ-9 | 34 | 0.025 | 0.066 | 0.040 (0.017 to 0.081) | 0.0 / 35.3 | 35.3 / 64.7 | 52.9 / 82.4 |
| GAD-7 | 20 | 0.171 | 0.200 | 0.030 (-0.046 to 0.100) | 60.0 / 65.0 | 70.0 / 80.0 | 80.0 / 95.0 |
| PSS | 492 | 0.577 | 0.916 | 0.338 (0.306 to 0.373) | 1.0 / 36.2 | 37.4 / 69.1 | 82.9 / 94.3 |
| ISI | 33 | 0.150 | 0.175 | 0.026 (-0.018 to 0.149) | 60.6 / 66.7 | 78.8 / 81.8 | 90.9 / 93.9 |
| PHQ-9 + GAD-7 + ISI | 87 | 0.082 | 0.119 | 0.037 (0.012 to 0.085) | 37.9 / 41.4 | 40.2 / 60.9 | 77.0 / 77.0 |

**Note:** Models were fitted separately within each analysis set. Several scales had small event counts and correspondingly wide uncertainty; PSS produced the largest and most stable increment.

**Supplementary Table S13.** Sensitivity of disagreement and HCD ranking to response-rich predictor sets.

| **Target** | **Model** | **AP** | **Delta AP (95% CI)** | **5% capture** | **10% capture** | **20% capture** | **30% capture** | **Events n** |
| --- | --- | --- | --- | --- | --- | --- | --- | --- |
| Ordinary disagreement | Response-rich | 0.540 | 0.000 | 9.5 | 18.0 | 34.4 | 49.0 | 2270 |
| Ordinary disagreement | Response-rich + RT | 0.540 | 0.000 (-0.003 to 0.002) | 9.4 | 17.8 | 34.1 | 48.5 | 2270 |
| Ordinary disagreement | Response-rich + shuffled RT | 0.538 | -0.002 (-0.003 to -0.001) | 9.6 | 18.1 | 34.3 | 49.1 | 2270 |
| HCD | Response-rich | 0.761 | 0.000 | 29.4 | 59.6 | 90.7 | 95.3 | 579 |
| HCD | Response-rich + RT | 0.769 | 0.007 (-0.001 to 0.017) | 29.9 | 60.3 | 90.7 | 95.5 | 579 |
| HCD | Response-rich + shuffled RT | 0.755 | -0.006 (-0.011 to -0.001) | 29.2 | 59.8 | 90.3 | 95.2 | 579 |

**Note:** Response-rich predictors included the exact selected-item mask, selected-item responses, confidence summaries, and score-and-coverage variables. These models ranked disagreements and did not replace the prorated score-category assignment.

**Supplementary Table S14.** Sensitivity to fixed-order and participant-specific item-selection designs.

| **Design** | **Target** | **Events n** | **AP C / C+RT** | **5% capture C / C+RT** | **10% capture C / C+RT** | **20% capture C / C+RT** | **30% capture C / C+RT** |
| --- | --- | --- | --- | --- | --- | --- | --- |
| strict observed first 19 | ordinary disagreement | 2616 | 0.696 / 0.735 | 6.9 / 7.1 | 13.5 / 14.2 | 27.3 / 27.8 | 38.7 / 39.7 |
| strict observed first 19 | HCD | 966 | 0.568 / 0.763 | 9.4 / 17.4 | 19.3 / 31.6 | 40.3 / 54.9 | 63.0 / 75.1 |
| post hoc near-balanced fixed-19 sensitivity (5/5/5/4 allocation; training-only item ranking) | ordinary disagreement | 2266 | 0.444 / 0.476 | 9.4 / 6.8 | 15.0 / 13.4 | 21.8 / 25.5 | 37.0 / 37.5 |
| post hoc near-balanced fixed-19 sensitivity (5/5/5/4 allocation; training-only item ranking) | HCD | 576 | 0.497 / 0.701 | 1.4 / 29.7 | 33.0 / 51.7 | 70.0 / 75.2 | 85.6 / 84.7 |
| participant-specific adaptive subset replay | ordinary disagreement | 2270 | 0.467 / 0.488 | 8.6 / 7.0 | 17.0 / 13.7 | 29.1 / 27.0 | 37.6 / 37.9 |
| participant-specific adaptive subset replay | HCD | 579 | 0.540 / 0.772 | 15.2 / 30.6 | 32.8 / 56.1 | 81.5 / 82.2 | 87.2 / 88.6 |

**Note:** HCD thresholds, event counts, and denominators were design-specific. Confidence-only and confidence-plus-RT models should be compared only within the same design; absolute capture rates do not provide direct comparisons of design quality.

**Supplementary Table S15.** Sensitivity of HCD definition and ranking to scale-specific validation thresholds.

| **Rule** | **Scale** | **Threshold** | **Ties n** | **HCD n** | **PSS share (%)** | **AP C / C+RT / shuffled** | **Delta AP (95% CI)** | **10% capture C / C+RT** |
| --- | --- | --- | --- | --- | --- | --- | --- | --- |
| >= threshold | PHQ-9 | 0.857675 | 112 | 215 | 55.0 | 0.213 / 0.271 / 0.184 | 0.057 (0.023 to 0.099) | 16.3 / 52.1 |
| >= threshold | GAD-7 | 0.660747 | 28 | 58 | 55.0 | 0.120 / 0.120 / 0.112 | 0.000 (-0.018 to 0.035) | 63.8 / 62.1 |
| >= threshold | PSS | 0.984648 | 105 | 390 | 55.0 | 0.587 / 0.921 / 0.435 | 0.334 (0.296 to 0.369) | 47.2 / 82.3 |
| >= threshold | ISI | 0.879030 | 1 | 46 | 55.0 | 0.261 / 0.306 / 0.261 | 0.045 (-0.035 to 0.149) | 82.6 / 84.8 |
| > threshold | PHQ-9 | 0.857675 | 112 | 103 | 61.6 | 0.133 / 0.150 / 0.122 | 0.017 (-0.017 to 0.070) | 34.0 / 48.5 |
| > threshold | GAD-7 | 0.660747 | 28 | 30 | 61.6 | 0.061 / 0.058 / 0.037 | -0.002 (-0.033 to 0.058) | 70.0 / 66.7 |
| > threshold | PSS | 0.984648 | 105 | 285 | 61.6 | 0.595 / 0.867 / 0.400 | 0.272 (0.225 to 0.325) | 64.6 / 89.8 |
| > threshold | ISI | 0.879030 | 1 | 45 | 61.6 | 0.265 / 0.332 / 0.269 | 0.067 (-0.025 to 0.186) | 84.4 / 88.9 |

**Note:** Thresholds were estimated separately from validation disagreements within each scale and applied unchanged to the corresponding test rows. Inclusive and strict rules are both reported because confidence values were discrete and ties were common. Total HCD count and PSS share are rule-level summaries and are repeated across scale rows to preserve one-row-per-scale machine-readable output.

**Supplementary Table S16.** Leakage-control and reproducibility checks for the offline adaptive replay.

| **Audit** | **Evidence** | **Status** |
| --- | --- | --- |
| Stepwise response perturbation | 475/475 prefix checks | PASS |
| Stepwise RT perturbation | 475/475 prefix checks | PASS |
| Full response perturbation | 8000/8000 policy simulations unchanged | PASS |
| Full RT perturbation | 8000/8000 policy simulations unchanged | PASS |
| Split identifiers | No overlap across train/validation/test participant IDs | PASS |
| RT residual model fitting | Participant IDs used to fit the RT residual models were a subset of the training IDs. | PASS |
| Fixed-prefix prediction invariance | 100 participants unchanged after future response/RT permutation | PASS |

**Supplementary Table S17.** Leave-one-scale-out transfer of disagreement-ranking models.

| **Held-out scale** | **Confidence only, AP** | **Confidence + RT, AP** | **Confidence + shuffled RT, AP** |
| --- | --- | --- | --- |
| PHQ-9 | 0.205 | 0.154 | 0.18 |
| GAD-7 | 0.642 | 0.082 | 0.385 |
| PSS | 0.484 | 0.39 | 0.473 |
| ISI | 0.243 | 0.158 | 0.223 |

**Note:** This leave-one-scale-out analysis evaluated cross-scale transfer of ordinary-disagreement ranking within the source dataset. Cross-scale transfer of HCD ranking was not evaluated.

**Supplementary Table S18.** Internal consistency of complete-questionnaire item responses.

| **Scale** | **Items** | **Cronbach's α** | **Scoring note** |
| --- | --- | --- | --- |
| PHQ-9 | 9 | 0.881 | Released score = sum of items |
| GAD-7 | 7 | 0.903 | Released score = sum of items |
| PSS | 14 | 0.793 | Released score retained; one reconstruction mismatch |
| ISI | 7 | 0.865 | Released score = sum of items |

**Note:** Cronbach's α is reported as a descriptive data-quality check. It does not establish diagnostic validity, unidimensionality, or the validity of the dataset-defined score categories.

**Supplementary Table S19.** Score-level sensitivity analysis using complete-questionnaire scores and near-boundary disagreement.

| **Analysis set** | **Score decisions, n** | **Full-score MAE** | **Median absolute score error** | **75th percentile absolute score error** | **Near-boundary decisions, n** | **Near-boundary decisions (%)** | **Near-boundary disagreements, n** | **Near-boundary disagreements (%)** |
| --- | --- | --- | --- | --- | --- | --- | --- | --- |
| PHQ-9 | 3644 | 0.888 | 0.8 | 1.2 | 1716 | 47.1 | 370 | 21.6 |
| GAD-7 | 3644 | 0.33 | 0.0 | 0.6 | 1055 | 29.0 | 31 | 2.9 |
| PSS | 3644 | 8.249 | 5.0 | 11.2 | 1538 | 42.2 | 851 | 55.3 |
| ISI | 3644 | 0.965 | 0.75 | 1.5 | 572 | 15.7 | 127 | 22.2 |
| PHQ-9 + GAD-7 + ISI (PSS excluded) | 10932 | 0.728 | 0.4 | 1.0 | 3343 | 30.6 | 528 | 15.8 |
| All scale decisions | 14576 | 2.608 | 0.857 | 2.0 | 4881 | 33.5 | 1379 | 28.3 |

**Note:** Full-score MAE and absolute score error use the released complete-questionnaire score as the reference. Near-boundary decisions had complete-questionnaire scores within two points of an operational category boundary. Near-boundary disagreement occurred when the 19-item estimate and complete score fell on opposite sides of at least one boundary. This analysis does not validate the diagnostic meaning of the thresholds.

# 2 Supplementary Figures

**Supplementary Figure S1.** Response-time residualization diagnostics. The left panel compares expected and observed log response time, and the right panel shows the content-adjusted residual distribution. The low training R² indicates limited explanatory power; the residuals were therefore treated only as operational preprocessing features rather than explanatory, causal, or latent-trait measures.

**Supplementary Figure S2.** Scale-specific reliability diagrams at the 19-item budget. Curves compare response-only screening with RT calibration-only probabilities for PHQ-9, GAD-7, PSS, and ISI. Bin counts are listed below each panel.

**Supplementary Figure S3.** Calibration-control comparison at the 19-item budget. Hatched bars indicate RT-based variants. The left panel reports label-aware expected calibration error (ECE-L) for every variant; the right panel reports Brier score.

**Supplementary Figure S4.** Short-form disagreement capture versus average questionnaire burden under selective escalation. The y-axis shows the percentage of short-form disagreements captured relative to complete-questionnaire score categories. The x-axis shows the average number of items completed as the participant-escalation rate increases.

**Supplementary Figure S5.** Mean elevated-score false-negative rate across the four scales by response-speed subgroup, with subgroups defined from the RT-aware 19-item selected set. This comparison is an exploratory process-audit diagnostic and was not subjected to formal inferential testing.

# 3 Supplementary Methods

## Supplementary Methods S1. Provenance and sensitivity of fixed item-selection parameters

Equation (16) defines a deterministic operational candidate-ranking utility that combines training-derived item priority, scale coverage, within-scale redundancy, and selected-item response-process consistency. Its coefficients place these components on comparable numerical scales and encode preferences for broad scale coverage, limited repeated selection within a scale, and additional confirmation using higher-priority items when selected-item RT patterns are less consistent. The coefficients were fixed during model development and were not selected using test outcomes. Supplementary Table S3 lists their values, operational roles, and data-access boundaries.

Supplementary Table S3 also documents two fixed constants used outside the item-selection utility. The item-priority score used variance weight $\omega_{I}= 0.05$, combining the absolute correlation between each item response and the corresponding complete-questionnaire score-category index with a small response-variance contribution. The label-preserving RT calibration-only control used $\gamma_{\mathrm{RT}}= 1.25$ to expand participant-specific smoothing as final-budget process consistency declined. Both constants were fixed during model development and retained unchanged for test-set evaluation. They served as operational constants and carried no standalone psychometric or clinical interpretation.

We examined the sensitivity of the main findings to the RT-modulation coefficients at the 19-item budget. These analyses were post hoc. A common multiplier $m\in\{0,0.5,1,2\}$ was applied to each RT-dependent increment:

$$\begin{aligned} U_{pst}\left( m \right)=U_{pst}^{\left( 0 \right)}\left[ 1+m\times0.25\left( 1-R_{pt} \right) \right],\#\left( S1 \right) \end{aligned}$$

$$\begin{aligned} \lambda_{pt}\left( m \right)=0.20+m\times0.40\left( 1-R_{pt} \right),\#\left( S2 \right) \end{aligned}$$

$$\begin{aligned} \rho_{pt}\left( m \right)=0.015+m\times0.020\left( 1-R_{pt} \right),\#\left( S3 \right) \end{aligned}$$

$$\begin{aligned} \eta_{pt}\left( m \right)=m\times0.25\left( 1-R_{pt} \right).\#\left( S4 \right) \end{aligned}$$

Values of $m$ were defined as follows: $m=0$ removed RT-dependent modulation, $m=0.5$ halved it, $m=1$ reproduced the reference RT-aware utility fixed during model development, and $m=2$ doubled it. The response-only base utility remained unchanged. We also removed each of five terms in turn: the RT uncertainty multiplier, the coverage term, the under-covered-scale bonus, the redundancy penalty, and the item-priority confirmation term. Each variant used the original participant splits and reran the full selection, score prediction, calibration, decision-audit, and escalation pipeline. Test performance was not used to select a variant.

Across the nine variants, mean macro-F1 ranged from 0.686 to 0.698, and ECE-L ranged from 0.095 to 0.099. Short-form disagreements ranged from 2,208 to 2,270, and HCD events ranged from 554 to 598. At 20% participant-level escalation, any-disagreement capture ranged from 25.6% to 29.8%, and HCD capture ranged from 76.1% to 82.5%. The reference RT-aware utility yielded a macro-F1 of 0.688, an ECE-L of 0.099, 2,252 disagreements, 598 HCD events, 26.5% any-disagreement capture, and 76.6% HCD capture. Classification and calibration changed little across variants. Audit ranking was more sensitive, particularly after coverage-related terms were removed. Some post hoc variants produced better test metrics; these results were interpreted as sensitivity findings and were not used to replace or retune the reference utility. Supplementary Table S4 reports the full results.

## Supplementary Methods S2. Target-matched HCD models and item-selection design-sensitivity analyses

The primary incremental comparison used two class-balanced L2 logistic HCD models with identical data splits, target definition, confidence features, C grid, validation selection, and train-plus-validation refitting. The augmented model added the RT summaries defined during model development. Participant risk was the maximum scale-level score. Paired participant-cluster bootstrap resampling estimated capture differences at 5%, 10%, 20%, and 30% participant capacity. Additional analyses evaluated disagreement within the fixed high-confidence gate, scale-specific and non-PSS models, response-rich non-RT features, strict observed-first-19 and near-balanced fixed-19 designs, and scale-specific validation thresholds. Each fixed-order design and threshold rule generated its own event set, so comparisons were interpreted within the corresponding design or threshold definition.
